# Low Quality of Free Coaching Apps With Respect to the American College of Sports Medicine Guidelines: A Review of Current Mobile Apps

**DOI:** 10.2196/mhealth.4669

**Published:** 2015-07-24

**Authors:** François Modave, Jiang Bian, Trevor Leavitt, Jennifer Bromwell, Charles Harris III, Heather Vincent

**Affiliations:** ^1^ University of Florida Department of Health Outcomes and Policy Gainesville, FL United States; ^2^ University of Florida Department of Orthopaedics and Rehabilitation Gainesville, FL United States

**Keywords:** apps, fitness, mHealth, mobile coaching, obesity, quality, weight loss

## Abstract

**Background:**

Low physical activity level is a significant contributor to chronic disease, weight dysregulation, and mortality. Nearly 70% of the American population is overweight, and 35% is obese. Obesity costs an estimated US$ 147 billion annually in health care, and as many as 95 million years of life. Although poor nutritional habits remain the major culprit, lack of physical activity significantly contributes to the obesity epidemic and related lifestyle diseases.

**Objective:**

Over the past 10 years, mobile devices have become ubiquitous, and there is an ever-increasing number of mobile apps that are being developed to facilitate physical activity, particularly for active people. However, no systematic assessment has been performed about their quality with respect to following the parameters of sound fitness principles and scientific evidence, or suitability for a variety of fitness levels. The aim of this paper is to fill this gap and assess the quality of mobile coaching apps on iOS mobile devices.

**Methods:**

A set of 30 popular mobile apps pertaining to physical activity programming was identified and reviewed on an iPhone device. These apps met the inclusion criteria and provided specific prescriptive fitness and exercise programming content. The content of these apps was compared against the current guidelines and fitness principles established by the American College of Sports Medicine (ACSM). A weighted scoring method based on the recommendations of the ACSM was developed to generate subscores for quality of programming content for aerobic (0-6 scale), resistance (0-6 scale), and flexibility (0-2 scale) components using the frequency, intensity, time, and type (FITT) principle. An overall score (0-14 scale) was generated from the subscores to represent the overall quality of a fitness coaching app.

**Results:**

Only 3 apps scored above 50% on the aerobic component (mean 0.7514, SD 1.2150, maximum 4.1636), 4 scored above 50% on the resistance/strength component (mean 1.4525, SD 1.2101, maximum 4.1094), and no app scored above 50% on the flexibility component (mean 0.1118, SD 0.2679, maximum 0.9816). Finally, only 1 app had an overall score (64.3%) above 50% (mean 2.3158, SD 1.911, maximum 9.0072).

**Conclusions:**

There are over 100,000 health-related apps. When looking at popular free apps related to physical activity, we observe that very few of them are evidence based, and respect the guidelines for aerobic activity, strength/resistance training, and flexibility, set forth by the ACSM. Users should exercise caution when adopting a new app for physical activity purposes. This study also clearly identifies a gap in evidence-based apps that can be used safely and effectively to start a physical routine program, develop fitness, and lose weight. App developers have an exciting opportunity to improve mobile coaching app quality by addressing these gaps.

## Introduction

### Background

Low physical activity levels significantly contribute to chronic disease, obesity, and all-cause mortality [1,2]. Since 2003, the prevalence of overweight and obesity in the United States has remained high [3]. As much as 68.5-75.3% of all adults 20 years of age or older are overweight or obese [3]. The annual health care burden attributable to obesity comprises 21% of the US health care expenditures [4]. The prevalence of overweight and obesity has not declined in the last decade [3,5], indicating that current strategies to address the problem in the general population have remained unsuccessful.

While it is known that increasing participation in regular exercise can help control body weight and reduce the risk of multiple comorbidities [2,6], it is estimated that only 20.6% of Americans actually meet the current recommendations of 2.5 hours minimum of moderate-intensity aerobic activity or 75 minutes of vigorous-intensity activity/week [7]. Among the barriers to exercise participation are the disparity in face-to-face access to health care professionals with expertise in lifestyle management, resources needed for a personal coach, and lack of knowledge of exercise principles necessary for someone to design their own training regimen [8-11]. Technological developments in the last 10 years have generated new strategies to broaden access to physical activity resources. Emerging evidence suggests that leveraging digital media may be an effective method to deliver health behavior interventions [12-14]. Electronic interface (Internet based) and mobile interface (either mobile phone or smartphone) are popular platforms [15,16]. Mobile phone and mobile phone ownership are accelerating quickly among young people and the general population [17]. Mobile technologies offer opportunities to mitigate the increasing disparity of access to affordable and even free resources [18,19]. There is an estimated 100,000 health care-related mobile apps [20]. Among these 100,000 apps, there is a fauna of apps to facilitate physical activity, including heart rate monitors, step counters, training logs, diet monitoring, and coaching.

### American College of Sports Medicine Guideline Overview

The American College of Sports Medicine (ACSM) [21] is the leading organization involved in the development and modification of exercise programming based on the cumulative evidence pertaining to exercise on health and fitness. The ACSM recommends that exercise programs should include key training elements of the frequency, intensity, time, and type (FITT) principle. Exercise programs should progress at a rate appropriate to the individual’s beginning fitness level and specific health or fitness goals. General exercise sessions typically include a warm up, conditioning and/or strengthening, a cool-down, and safety considerations. The successful translation of the ACSM guidelines into the mainstream of public use is dependent in part on the quality of information on the electronic media platforms accessed by the public. Presently, it is unclear whether the free mobile coaching apps available to the public provide adequate, accurate information of exercise programming for the beginning exerciser wishing to control body weight and manage comorbidities. Therefore, the goal of this investigation was to assess the quality of the most popular free health-related apps with respect to the general exercise program guidelines of the ACSM.

##  Methods

### App Selection Process

**Figure 1:**
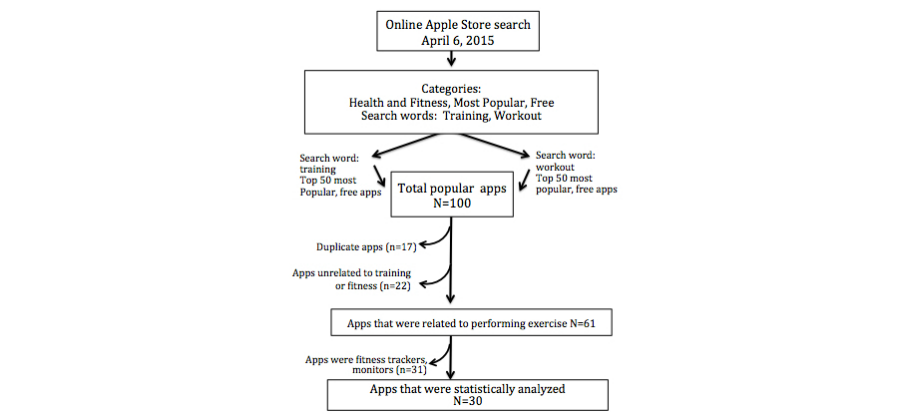
Flow diagram of iPhone coaching app selection.

As of 2014, Google Android (51.7%) and Apple iOS (38.9%) share over 90% of the mobile operating system market in the United States [22]. The choice of apps on both mobile operating systems reached 1.3 million and 1.2 million apps, respectively. However, popular apps are usually implemented cross-platform, and are therefore available on both app stores. Hence, our search was restricted to apps from the Apple store. For portability issues, we focused on apps that were available for iPhone, rather than other Apple devices such as iPad. Because we were primarily interested in assessing apps that could be used by the public without a financial burden, we restricted our search to apps that were available for free. Figure 1 illustrates our process of screening and selecting the relevant iPhone coaching apps for this analysis.

App stores are highly dynamic. Apps are added on a regular basis, and the ranking of popular apps changes weekly. The queries presented here were performed on April 6, 2015. We wanted to assess apps that would provide some workout or training programs. Thus, we selected the keywords “workout” and “training” and restricted our search to “health and fitness” free apps in the Apple store. A set of 50 apps was generated for each keyword term. The 2 sets of apps were merged. Because 17 duplicates existed, a list of 83 apps was generated. A prefiltering of the apps was performed by the study team to discard apps that were clearly not exercise prescriptive. The agreement among the study team was unanimous for discarding 22 apps from inclusion into the review. Examples of these irrelevant apps were for pregnancy, sleep quality, or menstrual cycle tracking. Of the 61 apps that were reviewed, 31 additional apps were discarded because exercise prescriptive programs were not provided, and therefore, did not meet the study inclusion criteria. The final set of 30 apps was scored for quality of content against the guidelines of exercise prescription of the ACSM.

### Principles of Exercise Prescription

The ACSM defines best practices in exercise prescription based on the known health benefits of exercise and physical activity. Optimal exercise programs include elements of cardiovascular fitness, endurance, strength, and flexibility, which collectively promote healthy body composition and neuromuscular fitness [21]. Exercise prescription consists of 3 main components, namely, aerobic exercise, strength and resistance exercise, and flexibility. Each component contains safety, programming (the FITT principle) and single-session principles. These components and principles are summarized in Multimedia Appendix 1.

Additional recommendations for safe exercise participation, particularly for beginners, include the following: reduce sitting time and sedentary behaviors, spread physical activity bouts throughout the day, no pain in joint, modify exercise in extreme environments (hot/humid), and be safe outdoors at night.

### App Quality Scoring Strategy

We developed our scoring strategy based on the recommendations set forth by the ACSM and our experience in assessing the quality of online weight loss information search results [23]. Therefore, each app was scored across the components of aerobic exercise, strength/resistance, and flexibility, with aerobic exercise and strength divided along the section of safety, program principles, and single training session principles, whereas flexibility was divided across safety and program principles only. There is no clear evidence that one of the 3 main components should be emphasized more than the other. Thus, we weighed the 3 individual component scores (endurance, strength, and flexibility) based on the time allocated by the ACSM within a standard exercise program for health and fitness. In addition, each section (ie, safety, program components, and single-training session components) of the 3 components was allocated the same weight due to the lack of evidence of one specific part being more important. For the same reason, each atomic criterion (FITT) was weighed identically. To allow further discrimination in the scoring, and to consider criteria that may be partially met, each criterion was scored as 0 (criterion not met), 1 (criterion somewhat met), and 2 (criterion met). Finally, the guidelines of the ACSM were operationalized in the following manner.

According to the ACSM guidelines, aerobic exercise safety is to be assessed with respect to 2 main criteria: the recommendation for physical examination before starting a program for populations at risk, and the recommendation for choosing an activity or activities that match a new exerciser’s skill levels. Therefore, safety was scored as “meets criterion” if both recommendations were made by the app’s designers, “somewhat met” if only 1 recommendation was made, and “not met” if neither was recommended. The safety of the strength/resistance component was assessed as “met” if the app did emphasize proper form and full range of motion when possible, with controlled breathing, “somewhat met” if controlled breathing or proper form/full range of motion was not emphasized, and “not met” if neither controlled breathing nor proper form was recommended. Finally, the safety component of flexibility was assessed in a similar manner with its 2 criteria being “no bouncing” and “light warm up prior to stretching.” Program principles and single-training session principles were scored in the same manner as safety, across all their atomic criteria, following the recommendations described in Multimedia Appendix 1. Table 1 provides the summary of the app quality scoring system developed and used by the study team. The range of possible points was 0-14.

While the evaluation of each criterion was relatively straightforward, each coaching app was scored independently by 3 team members using iPhone devices. Notes on the features of each app, limitations, and any unique features were collected from each team member for qualitative analysis. In the case of a discrepant finding, where one of the evaluators scored differently from the remaining 2, a fourth team member served as the arbitrator and scored the app to determine the score.

**Table 1:** Scoring system for the quality of the apps for exercise prescription and programming for beginners. The point value for each item is in parentheses.

App components	Aerobic exercise (score weight in points)	Strength/resistance (score weight in points)	Flexibility (score weight in points)
Safety	Safety (1)	Safety (1)	Safety (1)
Program principles	Frequency (0.1667)	Frequency (0.1428)	Frequency (0.1428)
	Intensity (0.1667)	Intensity (0.1428)	Intensity (0.1428)
	Time (0.1667)	Type (0.1428)	Time (0.1428)
	Type (0.1667)	Repetitions (0.1428)	Type (0.1428)
	Volume (0.1667)	Sets (0.1428)	Volume (0.1428)
	Progression (0.1667)	Rest (0.1428)	Pattern (0.1428)
		Progression (0.1428)	Progression (0.1428)
Single training session principles	Warm up (0.25)	Warm up (0.25)	
	Conditioning (0.25)	Conditioning (0.25)	
	Cool down (0.25)	Cool down (0.25)	
	Stretching (0.25)	Stretching (0.25)	
Possible points	6	6	2
		Total possible score (points)	14

### Statistical Analysis

Three reviewers evaluated the 30 apps during the last week of April 2015 and scored the apps using the scoring system shown in Table 1. An overall quality score and subscores for the 3 components of aerobic exercise, strength/resistance training, and flexibility were generated. Basic statistics were computed (arithmetic mean, SD, maximum for the set of the final 30 apps). A threshold quality score was established to indicate whether each app provided at least half of the content of the ACSM guidelines for the overall app score and the component subscores (3/6 points for aerobic exercise or strength/resistance components, and 1/2 points for flexibility). Inter-rater reliability was assessed using the Krippendorff alpha coefficient [24]. The results were visualized using box-plot mapping. Statistical analysis was performed using R software (version 3.1.2).

## Results

### Binary Evaluation

We initially looked at apps that met any of the recommendations of the ACSM guidelines to perform a first filtering of the results. Pertaining to the aerobic components, a bit more than half did include some of the recommendations. On the strength/resistance component, apps performed a bit better in the initial filtering phase with 90% (n=27) of them meeting at least one criterion. By contrast, they underperformed significantly on the flexibility component with two thirds of apps not meeting any criteria at all. These results are summarized in Table 2.

**Table 2:** Prevalence of apps that provided any information about the key components and principles of the ACSM guidelines, 9th edition.

Program component	Met “any” of the key components of the ACSM	Met “none” of the key components of the ACSM
Aerobic	17 (56.6%)	13 (43.4%)
Strength/resistance	27 (90%)	3 (10%)
Flexibility	10 (33.3%)	20 (66.7%)

### App Quality Score

The final 30 apps included in this review are listed in Table 3, where apps are presented in order from the highest to lowest average app quality score. The maximal points for the overall quality score was 14 points; the maximal aerobic and resistance component quality subscores were 6 points and the flexibility subscore was 2 points.

**Table 3:** Training and workout apps included in the study analysis.

App	Overall quality Score (points)	Quality subscores (points)^a^	App comments
Sworkit Lite Personal Trainer	9.01	3.92; 4.11;0.98	Provides good variation for 30-minute workouts, all 3 workout components and programming are present
The 7 Minute Workout-Get fit	5.39	2.53; 2.86; 0.00	Provides aerobic and resistance workouts, but no fitness program elements and progression
StrongLifts 5x5	4.47	0.99; 3.48; 0.00	Provides workout plan and progression; mainly focused on strength training
Running for Weight Loss: Interval Training	4.16	4.16; 0.00; 0.00	Provides a running training plan with all aerobic program elements, but no resistance training/flexibility instruction
JEFIT Workout	4.08	0.47; 3.51; 0.10	Provides workouts and video examples. Program elements are present, but must be set by the user
FitnessBuilder	4.04	0.99; 2.88; 0.17	Provides exercises for various body parts but no program elements; need upgrade to progress beyond beginner workouts
C25K-5K Trainer Free	3.67	3.67; 0.00; 0.00	Provides general progression plan to work up to running continuously for 4.8 km (3 mi)
Ultimate Fitness Free	3.53	0.00; 3.53; 0.00	Provides some workouts, payment required to access all features, but no fitness program components
Nike+ Training Club	3.11	1.14; 1.92; 0.05	Social network and workout log
BodySpace	2.56	0.00; 2.31; 0.25	Substantial index of exercises for workouts, workouts can come from different coaches, has pictures of exercises and demonstrations
Fitness Buddy Free	2.53	0.50; 2.03; 0.00	Provides workout videos and individual workout plans; fitness program elements and progression; targeted for women; 1 workout provided free
7-Minute Workout-Fitness for Women	2.54	1.13; 1.41; 0.00	Provides aerobic and resistance workouts, but no fitness program elements and progression; targeted for women; 1 workout provided
The Johnson and Johnson Official 7-Minute Workout	2.44	1.22; 1.22; 0.00	Provides aerobic and resistance workouts, but no fitness program and progression elements
Fitness Point-Workout Exercise	2.05	0.00; 2.05; 0.00	Provides strength training sessions only
FitStar Personal Trainer	1.99	0.48; 1.47; 0.04	Provides log for workouts and workouts to do, provides a few workouts for free but all program features only available with subscription; good exercise demonstrations and videos
7 Minute Workout	1.72	0.00; 1.72; 0.00	Provides some workouts, payment required to access all features, but no fitness program components; good basic fitness program
Instant Abs Trainer	1.64	0.00; 1.64; 0.00	Provides exercises for all body parts, but contains no fitness program components
Daily Workouts Free	1.56	0.05; 1.44; 0.07	Provides workout exercises and video, but no fitness program components; a “a home-made video” presentation
Jillian Michaels Slim Down	1.43	0.11; 1.27; 0.05	Features exercises for all body parts, but access to program details must be purchased by consumer
Simply Yoga Free	1.38	0.33; 0.33; 0.72	Only focuses on yoga; 1 free workout-only reduction; different workouts based on experience, track progress with pictures
Belly Fat Workout Free	1.38	0.00; 1.38; 0.00	Workouts geared to burning abdominal fat, “spot training”
Daily Yoga-Lose Weight, Get Relief	1.26	0.00; 0.33; 0.93	Provides exercises but no fitness program components
Cardio-Heart Rate Monitor + 7 Minute Workout	0.66	0.33; 0.33; 0.00	Heart rate monitor and tracker; has 7-minute aerobic and resistance workouts to improve fitness and endurance but no progressive fitness programming
Daily Butt Workout FREE	0.55	0.00; 0.55; 0.00	Provides exercises for all legs and glutes, but contains no program elements. Paid version includes more exercises for various body parts
Strava Running and Cycling	0.49	0.49; 0.00; 0.00	Provides workouts and log for workouts; only tracks when premium is purchased by consumer
Workout Trainer	0.44	0.00; 0.44; 0.00	Provides exercises for multiple body parts and logging tools. No fitness program components provided
8Fit Fitness at Home: Personal Trainer	0.33	0.00; 0.33; 0.00	Provides workouts and workout programs, but program components and progression features are only available after payment
Daily Ab Workout Free	0.33	0.00; 0.33; 0.00	Only an abs workout trainer, not other components
Abs Workout: Get Your Six Pack	0.33	0.00; 0.33; 0.00	Only an abs workout trainer, not other components
Runtastic Six Pack Abs Trainer	0.33	0.00; 0.33; 0.00	Only an abs workout trainer, not other components

^a^Quality subscores are for aerobic; resistance; flexibility.

**Figure 2:**
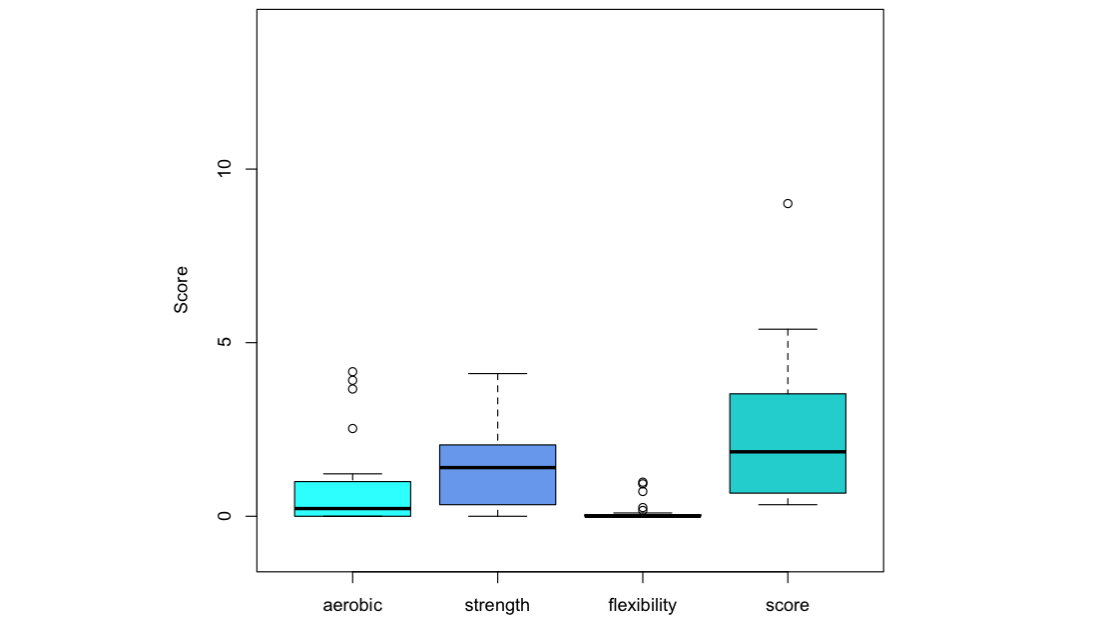
Box plot of app quality scores and subscores relative to the American College of Sports Medicine recommendations of exercise prescription and programming. Higher scores represent higher app quality.

Among the 30 apps, only 3 apps (Sworkit Lite Personal Trainer, C25K-5K Trainer Free, Running for Weight Loss: Interval Training) scored above 50% on the aerobic component on a 0-6 scale (mean 0.7514, SD 1.2150, maximum 4.1636). Four apps (Sworkit Lite Personal Trainer, Ultimate Fitness Free, JEFIT Workout, and StrongLifts 5x5) scored above 50% on the resistance/strength component on a 0-6 scale (mean 1.4525, SD 1.2101, maximum 4.1094). Finally, none of the apps scored above 50% on the flexibility component on a 0-2 scale (mean 0.1118, SD 0.2679, maximum 0.9816). Finally, only 1 app (Sworkit Lite Personal Trainer) had an overall score (64.3%) above 50% on a 0-14 scale (mean 2.3158, SD 1.911, maximum 9.0072). The results are summarized in Table 4 and in the box plot of app quality scores and subscores with respect to the ACSM recommendations (Figure 2). The inter-rater reliability statistics on the overall app score broken down by component (aerobic, strength, and fitness) is .636 (Krippendorff alpha). The alpha is rather low (<.800), which indicates a low agreement among the 3 reviewers on the exact score. However, the 3 reviewers did agree on the fact that none of fitness and workout apps on the market meet all of the ACSM exercise prescription guidelines.

**Table 4:** Basic statistics of app quality scores based on the American College of Sports Medicine exercise prescription guidelines.

	Aerobic component	Strength component	Flexibility component	Overall quality score
Mean	0.7514933	1.452537	0.1118478	2.315878
SD	1.215081	1.210152	0.267902	1.911409
Maximal score	4.1636	4.109467	0.9816	9.0072

##  Discussion

### Principal Findings

Despite the relatively large number of fitness and workout apps, our findings indicate that very few of them are of sufficient quality to provide evidence-based exercise prescription, especially for beginners.The results are rather striking. Barely 20% (n=6) of the most popular free apps attained the quality threshold score of 50% for 1 subscore, and only 1 app scored above 7/14 points. During the scoring process, we collected comments from the study team on the apps. The most frequent criticism reported by the study team in 23 of 30 apps was that the apps did not provide an actual training plan, explaining how to choose the workouts and how to organize them in a week, although specific training sessions were provided. Only 4 provided training plans followed a safe and physiologically sound progression. Thus, a significant gap exists in available mobile coaching app technology, especially for novice exercisers. As such, there is the risk for users to participate in exercise programs without the appropriate level of physical preparedness, technique, and awareness of safety concerns.

**Figure 3:**
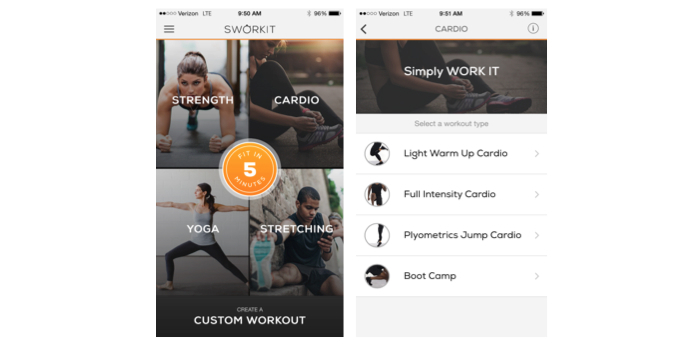
Sworkit app screenshots.

Key features of the 2 highest quality apps include components of the training programs, exercise instruction, and a variety of activity within exercise sessions. The 2 top scoring apps were “Sworkit Lite Personal Trainer” and “7 Minute Workout.” “Sworkit Lite Personal Trainer” was the most comprehensive app that scored 9 points out of a possible 14 points and covered all 3 ACSM components of a well-designed training program. However, some of the exercises described may be technically difficult for beginners, such as plyometrics, which are associated with higher rates of injuries compared with nonstretch and activation movement [25,26]. A strength of this app was a good variation of exercises for different 30-minute workout sessions (Figure 3).

**Figure 4:**
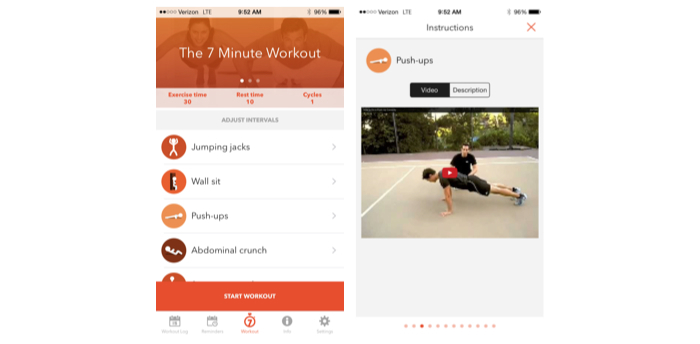
7 Minute Workout app screenshots.

The “7 Minute Workout” app from Get fit had an overall quality score of 5.38 points of a possible 14 points (Figure 4 shows a screenshot of this app). The exercises were well structured and well explained, even for novice exercisers. Useful video demonstrations were helpful for skill development and safe exercise execution. However, the app lacked the key elements of a fitness program, such as frequency, duration, intensity, and training progression

Several other apps had relatively high-quality content in 1 component, but not all. For example, “StrongLifts 5x5” and “Running for Weight Loss: Interval Training” had high-quality subscores for resistance exercise (3.48 points) and aerobic exercise (4.16 points), respectively. The other subscores for these apps were low or 0.00 points. Six apps were designed specifically to target 1 body part or area, such as the buttocks or abdomen, and did not address overall musculoskeletal, aerobic, or flexibility fitness (Runtastic Six Pack Abs Trainer, Abs Workout: Get Your Six Pack, Daily Ab Workout Free, Daily Butt Workout FREE, Belly Fat Workout Free, and Instant Abs Trainer). While each of these apps possesses certain value for guiding users on specific types of exercise or to meet targeted goals, these are not effective coaching apps for improving overall fitness in a manner endorsed by the ACSM. Most of the apps provided in Table 3 were aesthetically pleasing and interesting to view. However, several key considerations of the user were not typically taken into account including the initial fitness level, age, skill level, or familiarity with the exercise type (yoga, heavy lift maneuvers, or running technique) and exercise preferences. Most of these apps did not provide a live social element that could boost exercise participation rates, such as social messaging. Importantly, many apps are not free, which is a barrier to users. Even among the free apps provided here, some provided partial content, and the full benefits of the app could only be attained after payment or subscription, such as “Jillian Michaels Slim Down” and “FitStar Personal Trainer.” The challenge for the general user is to sift through the hundreds of “free” available apps to determine what would be most useful, instructive, and safe to follow.

### Limitations

**Figure 5:**
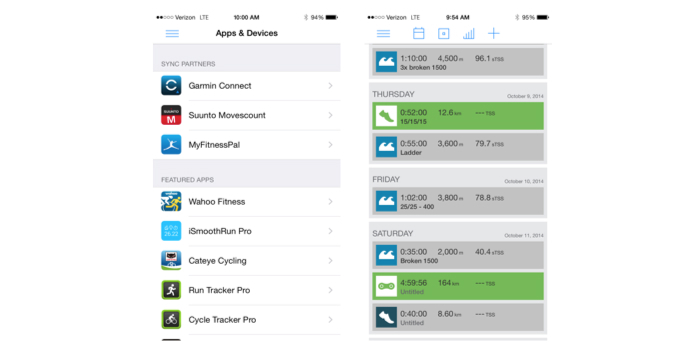
TrainingPeaks app screenshots.

There are some limitations to this work that deserve comment. As we noted, our scoring process is derived directly from the time recommendations of the ACSM on aerobic exercise, strength/resistance, and flexibility. The quality scores are based on the assumption that all the atomic elements of each component contribute equally to an athletic training program. This may not be the case. However, this is the most rational choice in the absence of evidence. There is the potential that an automatic query program over time may have yielded some additional apps that might have fit our inclusion criteria. However, we consider this a small possibility given that the content and complexity of the training and workout apps have been steadily improving over the last year. Additional queries over time would provide important insight into the evolution of these training and workout apps and how users value specific content. In addition, popular apps remain rather stable over relatively short periods. Another limitation of our study is that we restricted ourselves to free apps. However, an informal browsing of paying apps suggested that they do not score much better overall, with the inclusion of coaching features, personal adjustments to the training program, and additional variations of workout sessions. A few exceptions exist. For instance, TrainingPeaks was developed primarily by coaches and exercise physiologists. It provides desktop and mobile platforms for logging workouts (running, cycling, triathlons, and strength workouts), training articles, a variety of metrics to track performance, ways to upload training data, and also training plans, for a varying fee. However, it is probably too complex for beginners, and its fees may be intimidating, and a barrier for many users (Figure 5). Last, the inter-rater reliability statistics (computed using Krippendorff alpha) is rather low (alpha=.636) in this study, which renders the necessity of a future study to refine the instrument and evaluate its test-retest reliability. One potential reason for a lower-than-expected agreement coefficient is reviewers’ interpretations of the ACSM guidelines. However, our study is the very first that aimed to evaluate fitness and workout apps with respect to ACSM exercise prescription guidelines.In addition, all reviewers do agree on the fact that none of the apps we reviewed met all 3 components of the ACSM guidelines.

### Comparison With Prior Work

To the best of our knowledge, no work has previously evaluated the quality of mobile apps that are exercise prescriptive in nature. Some work [27-30] has been performed on the characterization of health and fitness-related apps, presenting the app functionalities, and studying health behavior theory constructs in these apps. These previous works have not assessed the app quality with respect to well-established guidelines of exercise prescription.

### Need for Evidence-Based, Accessible Mobile Coaching for Public Use

These data strongly support the needs of developing new mobile fitness apps that adhere to the ACSM guidelines for exercise prescription for use in the general public. Scientifically sound fitness programs are needed to increase physical activity among Americans, and more generally for public health.

Free mobile coaching apps may be a technology vehicle for directly translating ACSM guidelines into changing the activity behavior of public in a safe, scientifically sound manner. The popularity of mobile apps may make exercise and physical activity more appealing to different age groups. Moreover, the use of apps may remove disparities of access to free resources for health improvement. If effective, these apps may become an important component of primary care and disease prevention plans.

The results indicate that developers of mobile coaching apps have a unique opportunity to make a considerable impact in the field of health and fitness. Incorporation of the ACSM guidelines and FITT principles into app platforms using easy-to-understand language, pictures and videos, and progress trackers for exercise progression is critical to help users participate in regular activity. Moreover, platforms that contain educational pearls and answer questions (“Should I feel this burning ache in my muscle after I perform a set of chest press exercise?” or “Is it normal for me to breathe really hard and feel my heart pumping very fast when I run hard?”) can guide the exerciser about what to expect. Engagement of the user in the exercise process with the app may help improve adherence, reduce anxiety about the exercise experience, and empower the exerciser to progress further in his/her program by improving self-efficacy. Given the lack of safety considerations provided in current apps, app developers can immediately improve app quality by including key information such as contraindications to exercise, when to see a doctor before starting an exercise program, what pain is normal and what is not, and what precautions should be taken when exercising in hot weather or outside in the dark. Finally, coaching apps should be flexible with respect to individual preferences and availability to exercise equipment. Accounting for user exercise-type preferences is essential for exercise consistency and adherence over the long term.

### Conclusions

The study team analyzed the content of 30 apps that met the inclusion criteria and were considered exercise prescriptive. These apps were scored with respect to the quality of information provided relevant to the current guidelines of the ACSM. Nearly all the apps, although technically well designed, did not meet the basic recommendations of the ACSM for exercise prescription, and therefore, would not be suitable for beginning exercisers. Free apps designed with the 3 key components of ACSM exercise programming following the FITT principle, safety, and individual session structure are desperately needed for public use. These apps can be the basis for setting and safely achieving fitness, body weight, and health goals.

## Appendix

Multimedia Appendix 1 American College of Sports Medicine exercise program guideline summary for health and fitness.

## Abbreviations

ACSM: American College of Sports Medicine
FITT: frequency, intensity, time, and type
