# Supplementary material for: Low Quality of Free Coaching Apps With Respect to the American College of Sports Medicine Guidelines: A Review of Current Mobile Apps
Source: JMIR Mhealth Uhealth. 2015 Jul 24;3(3):e77. doi: 10.2196/mhealth.4669 (PMC4529492; doi:10.2196/mhealth.4669)
Supplement: Multimedia Appendix 1 [file mhealth_v3i3e77_app1.pdf]

Section	Aerobic exercise	Component<br>Strength/resistance	Flexibility
Safety	Physical examination by physician for men over 45 and women over 55, history of heart disease in family, smoker or recent former smoker, have not exercised regularly in last 3 months, overweight or obese, high blood pressure or cholesterol, prediabetic. Select activities that match your skills, balance, and strength.	Perform with proper technique, full range of motion in joint when possible, controlled breathing during the concentric and eccentric phases.	No bouncing while holding a stretch. Performed after a light warm up with aerobic activity or with heat packs to enhance stretching benefits.
Program principles	*Frequency*: 3-5 times/week<br>*Intensity*: HR methods, VO_2 methods, or perceived exertion methods.<br>*Time*: 20-60 minutes a day of vigorous activity or 30-60-minute moderate intensity/day 150 minutes or more of activity a week of moderate intensity 75 minutes or more of activity a week of vigorous intensity.<br>*Type:* Requiring minimal skill, especially for vigorous activity (eg, walking, cycling, running),	*Frequency:* Each major muscle group trained 2-3 times/week<br>*Intensity:* 60-70% of 1 repetition maximum for novice to intermediate, higher than 80% for experienced, 40-50% for older beginners and sedentary people beginning strength training.<br>*Type:* Multijoint exercise activating agonist/antagonist groups for all. Single-joint exercises can be included. Variety of machines, free weights, body weights.<br>*Repetitions:* 8-12 to improve strength for most.<br>*Sets:* 2-3.	*Frequency:* 2-3 times/week<br>*Intensity:* Stretch to point of tightness, no pain.<br>*Time:* Static stretch 10-30 seconds for most adults, 30-60 in older adults. For proprioceptive neuromuscular facilitation stretching: 3-6-second light/moderate contraction then stretch for 10-30 seconds.<br>*Type:* Series of stretches of each of the major muscle tendon regions are recommended.<br>*Volume:* Total stretch

	recreational sports.<br>*Volume:* Target 500-1000 metabolic equivalent of task/minute/week.<br>*Progression:* Increase time 5-10 minutes/session every 1-2 weeks	*Rest:* 2-3 minutes between sets and 48 hours between days.<br>*Progression:* Gradual/week	time of 60 seconds per muscle-tendon region<br>*Pattern:* Repeat stretching 2-4 times<br>*Progression:* Unknown
Single training session principles	*Warm-up:* 5-10 minutes of light/moderate intensity cardiovascular exercise.<br>*Conditioning:* 20-60-minute aerobic, resistance, neuromotor, or sport activities; accumulative bouts of 10 minutes of activity<br>*Cool-down:* 5-10-minute light/moderate cardiovascular exercise<br>*Stretching:* 10 minutes or more after exercise	*Warm-up:* 5-10 minutes of light/moderate intensity cardiovascular exercise.<br>*Conditioning:* Exercises<br>*Cool-down:* 5-10 minutes of light/moderate intensity cardiovascular exercise.<br>*Stretching:* 10 minutes or more after exercise.	
